# P51. Identification of prostate cancer-associated antigens by oxygen manipulation

**DOI:** 10.1186/2051-1426-2-S2-P25

**Published:** 2014-03-12

**Authors:** T Ma, A Elkhattouti, F Kosari, RJ Karnes, JC Cheville, G Vasmatzis, S Vuk-Pavlovic, RC Gomez

**Affiliations:** 1 University of Mississippi Medical Center, Cancer Institute, Jackson, USA; 2 Mayo Clinic, Molecular Medicine, Rochester, MN, USA; 3 Mayo Clinic, Urology, Rochester, MN, USA; 4 Mayo Clinic, Laboratory Medicine and Pathology, Rochester, MN, USA; 5 Mayo Clinic, Cancer Center and Internal Medicine, Rochester, MN, USA

## Background

Therapeutic vaccination against prostate cancer (CaP) remains marginally effective. A reason for failure may stem from the fact that vaccine cells are usually cultured in ordinary air. Solid tumours including CaP contain regions with oxygen deficiency (hypoxia) secondary to the lack of blood supply to the growing tumour nodules, which may lead to changes in expression of cancer-associated antigens in tumour cells. To test this hypothesis we determined whether oxygen-sensitive CaP-associated antigens in cultured CaP cell lines and human tumour tissues exist.

## Material and methods

LNCaP and VCaP prostate cancer cells were propagated in culture media conditioned by the cells at normoxic (20% O_2_) and hypoxic (2% O_2_) environment. At first, we measured release of vascular endothelial growth factor (VEGF) by ELISA and the expression of VEGF-α mRNA by RT-PCR. To identify potential CaP-associated antigens, we prepared CaP cell lysates, resolved them by 2D electrophoresis and immunoblotting using spontaneous antibodies from plasma derived from CaP patients and control subjects. Antibody-labelled spots were analysed by MALDI-TOF mass spectrometry. Furthermore, we evaluated the expression of selected candidates in native CaP tissue.

## Results

Hypoxic CaP cells released more VEGF (P<0.05) and expressed more mRNA for VEGF-α (p<0.001) than normoxic cells. After two days of culture, hypoxic cells expressed some forty fold higher amount of VEGF transcripts compared to normoxic cells. CaP-associated spots identified in this study included heat shock protein 70 (HSP70), HSP60 and heterogeneous nuclear ribonucleoprotein L (hnRNP L). Among them, HSP70 and hnRNP L were O_2_-sensitive. Level of the two proteins were two times higher in CaP tissue than in control benign prostate tissue (p<0.05).

## Conclusion

A unique set of O_2_-sensitive CaP-associated antigens exist in CaP tumour tissues and spontaneous antibodies are detected in plasma derived from CaP patients. Therefore, CaP cells grown at hypoxic condition may provide a better antigen match to tumours *in situ* and may be more effective vaccines. (Supported by UMMC Incentive (to TMA), DOD PC094680 and PCF Creativity Award (to CRG).

